# Significance of Postprandial Insulin and Triglycerides to Evaluate the Metabolic Response of Composite Meals Differing in Nutrient Composition – A Randomized Cross-Over Trial

**DOI:** 10.3389/fnut.2022.816755

**Published:** 2022-03-02

**Authors:** Rohith N. Thota, Paul J. Moughan, Harjinder Singh, Manohar L. Garg

**Affiliations:** ^1^Riddet Institute, Massey University, Palmerston North, New Zealand; ^2^Nutraceuticals Research Program, School of Biomedical Sciences & Pharmacy, University of Newcastle, Callaghan, NSW, Australia

**Keywords:** post-prandial insulin, post-prandial triglyceride, composite meals, macronutrient, nutrition profile

## Abstract

**Background and aims:**

GlucoTRIG, based on postprandial plasma insulin and triglyceride concentrations, has been recently developed as a novel index to determine the postprandial metabolic response to the meals. This study aimed to test GlucoTRIG as a measure for ranking composite meals for their metabolic effects.

**Methods:**

In a randomized cross-over trial, healthy adult volunteers (both males and females; *n* = 10 for each meal) consumed three is caloric (2000 kj) test meals (meal 1, meal 2, meal 3) of varying macronutrient composition. Postmeal consumption, venous blood samples were collected to determine plasma insulin and plasma triglycerides for estimating the GlucoTRIG value using (Triglycerides_180min_ × Insulin_180min_) - (Triglycerides_0min_ × Insulin_0min_).

**Results:**

The GlucoTRIG values differed significantly (*p* = 0.0085) across meals. The statistical significance remains even after adjusting for confounding variables such as baseline diet, insulin, and triglycerides. The meal (M3) with a high fiber, low total fat content and containing less refined foods (fruits, beans, vegetables, plain yogurt) exhibited a significantly (*p* = 0.007) lower GlucoTRIG value (10 ± 7.7) compared to the other two meals, M1 (77 ± 19.8) and M2 (38 ± 12.1) which contained low processed foods, and were relatively high in fat and low in fiber meals. No statistically significant differences were observed between M1 and M2 meal.

**Conclusions:**

GlucoTRIG is a physiologically based index that may be useful to rank composite meals for reducing the risk of metabolic diseases. Further research focusing on the application of GlucoTRIG to foods, meals, and diets is warranted.

ACTRN12619000973112 (Australian New Zealand Clinical Trials Registry, ANZCTR).

## Introduction

The epidemic of metabolic diseases has encouraged the development of nutrient profile models in an attempt to improve people's understanding of healthier foods to prevent obesity, type 2 diabetes (T2DM), and cardiovascular disease (CVD) (1). Established physiological models applied to meals and single foods, have substantial scope to assist consumers to understand the nutrient composition of foods and their metabolic effects in a better way (1). A better understanding of the relationship between diet and health has increased the demand for food nutrient indices utilizing postprandial measurements. The utilization of fasting metabolic parameters, such as plasma glucose and triglycerides, for clinical diagnoses is well-established, however, most people are predominantly in the postprandial state (nearly 16–18 h per day). Recent data provide evidence for postprandial lipid, glucose, and insulin abnormalities as independent risk factors for nutrition-related non-communicable diseases (2, 3). Postprandial hyperglycemia raises the risk for T2DM (3), CVD and cardiovascular mortality, even in individuals with fasting glucose levels within acceptable ranges (4). Available evidence also suggests that the postprandial triglyceride level is an independent predictor of CVD (5). These observations highlight the relevance of diet and its metabolic consequences for the risk of developing metabolic diseases.

Nutrient indices that have been previously developed are based on either the type or amount of a single macronutrient. These indices reflect the bio-availability of certain nutrients and their potential effects on metabolic parameters, to determine the healthiness of foods and meals (6). However, meals containing single nutrients are rarely consumed; instead, snacks or meals with multiple macronutrients are consumed in daily diets. The metabolic response of a meal depends on the relative proportion of the macronutrients (7). It has been widely assumed that blood insulin response is proportional to blood glucose levels and therefore has not been utilized independently in the nutrient indices. Protein and fat-rich foods may induce substantial insulin secretions despite producing relatively small blood glucose responses (8–10). Adding a high amount of fat to carbohydrate-rich meals can reduce the glucose response (11). Further, the amount of fat in a meal, along with the fatty acid composition, and can have profound effects on blood lipid and lipoprotein concentrations (12) and circulating insulin levels and insulin sensitivity (13, 14). Though there is evidence that high-fat diet intake is associated with both insulin resistance and post-prandial metabolic abnormalities (15), fat is mainly neglected when determining the healthiness of foods and meals.

We have recently introduced and conceptualized GlucoTRIG index, that incorporates the effects of triglycerides via serum triglyceride change, and that of glucose and other macronutrients and their combined effect via insulin measurement, to determine the metabolic response to a meal. We hypothesize that meals with high GlucoTRIG values have high post-prandial glycaemic and lipaemic responses, and the index is therefore assumed to be related to the healthiness of the meals or diets. We have evaluated a standard comparator (reference meal comprising of equivalent carbohydrate and fat proportions (41 and 40% of the total energy) for testing meals in our previous study (16). The GlucoTRIG reference meal has been shown to give acceptably low between-meal variation for the index values and can be used in future studies as a standard comparator to rank the GlucoTRIG values of meals. In the current study, we aimed to systematically evaluate GlucoTRIG values for these meals with different macronutrient contents expressed relative to an iso-caloric reference meal in a randomized cross-over trial with healthy subjects.

## Methods

### Subjects

Participant characteristics have been described in detail previously (16). Participants were recruited from the University of Newcastle, New South Wales, Australia, between September and November 2018. All the participants met the study inclusion criteria; aged between 18 and 40 years at initial assessment, body mass index (BMI) between 18 and 29.9 Kg/m^2^, non-smokers; not pregnant; currently not taking any lipid-lowering drugs or supplements (e.g., statins, fish oil) or anti-hypertensive drugs; no history of eating or metabolic disorders; no allergy or intolerance to any of the food products or ingredients used in the study. All participants gave written informed consent. The study protocol was approved by the University of Newcastle Human Research Ethics Committee (H-2016-0315), was registered with the Australian New Zealand Clinical Trials Registry (ACTRN12619000973112).

### Reference Meal

The detailed nutrient composition and GlucoTRIG value of the reference meal has been previously published (16). The reference meal consisted of two regular slices (28 g) of wholemeal bread (Woolworths select, Newcastle, Australia), 250 mL chocolate flavored milk (OAK Parlamat, Newcastle, Australia), 7 g unsalted butter and 11 g peanut butter (Kraft Foods Inc. Melbourne, Australia) delivering carbohydrates (41%), fat (40%), and protein (16%) of the total energy content (2000 kj) of the meal.

### Test Meals

Three separate test meals consisting of common foods: meal one (M1) consisted of Nutella (20 g, consisting of sugar, milk, palm oil, cocoa, hazelnuts, lecithin, and vanilla) manufactured by Ferrero Australia Pty Ltd., whole-grain seed toast (two slices consisting of whole grain whole meal wheat flour, mixed whole grain, wheat gluten, mixed seeds, kibbled soy, canola oil, baker's yeast, vinegar, iodized salt, cultured whey, buckwheat, corn) manufactured by George Weston Foods, 250 mL Up&Go iced coffee consisting of filtered water, skim milk powder, soy protein, cane sugar, maltodextrin (wheat, corn), milk protein concentrate, vegetable oils, fructose (Sanitarium, Australia); meal two (M2) comprised 180 g instant oats (Uncle Tobys, Australia), 250 mL skim milk, 1 medium banana and raw almonds (9 nuts); meal three (meal3, M3) provided 300 g watermelon, 200 g carrot slices, 170 g red kidney beans, 170 g plain yogurt (Chobani Australia Pty Ltd.,) and 250 ml tomato juice (Berri, Australia). All the meals were caloric (2000 kj), and the nutrient composition of the meals is presented in Table 1. Of the total energy content (2000 kj), M1 delivered carbohydrates (42%), fat (29%) and protein (20%); M2 delivered carbohydrates (56%), fat (23%) and protein (17%); while M3 delivered carbohydrates (58%), fat (4%), and protein (23%) (Table 1).

**Table 1:** Macronutrient composition of test meals and reference meal.

**Food category**	**Serving size**	**Total carbohydrate (excluding fiber)**	**Sugars**	**Starch**	**Total fat**	**Saturated fat**	**Polyunsaturated fat**	**Monounsaturated fat**	**Dietary fiber**	**Protein**
	**(g)**	**(g)**	**(g)**	**(g)**	**(g)**	**(g)**	**(g)**	**(g)**	**(g)**	**(g)**
**Reference meal** Wholemeal bread^1^ Butter^2^ Peanut butter^3^ Chocolate milk^4^	339	50	26	24	22	11	1	8	6	19
% energy		41			40				2	16
**Test meal 1 (M1)** Ferrero Nutella^5^ Hazelnut Spread Wholemeal & Seeds bread^6^ Up&Go Energize Iced Coffee Flavor^7^	348	60	31	29	16	3	7	6	10	25
% energy		42			29				4	20
**Test meal 2 (M2)** Oats Quick Sachets^8^ Original Almond, raw nuts^9^ Skim milk^10^ Banana^11^	385	67	24	43	13	2	3	7	10	20
% energy		56			23				4	17
**Test meal 3 (M3)** Greek yogurt plain^12^ Watermelon^13^ Red kidney beans^14^ Carrot^15^ Juice, tomato^16^	1,140	72	50	21	2	0	1	0	21	27
% energy		58			4				8	23

*^1^2 slices (28 g each), Woolworths select; ^2^7 g portion, Western Star; ^3^11 g portion, Kraft smooth peanut butter; ^4^250 mL OAK chocolate milk, Parmalat; ^5^20g, Nutella; ^6^2 slices, Burgen wholemeal and seeds toast; ^7^250 mL, Sanitarium up&go energize coffee flavor; ^8^2 sachets, Uncle Tobys; ^9^9 nuts, raw almond, Woolworths select; ^10^200 mL Devondale Skim milk; ^11^1 medium Cavendish banana; ^12^170 g, Chobani greek yogurt plain fat free; ^13^300 g, Woolworths; ^14^170 g, Edgell red kidney beans (drained); ^15^200 g raw unpeeled carrots, Woolworths; ^16^300 mL plain tomato juice. All the meals provided 2000 kj of energy based on the tabulated values. Data is obtained using FoodWorks software*.

### Study Design and Test Day Protocol

A randomized, cross-over trial was conducted on three separate days with a washout period of at least three days between each study day. All participants were randomly assigned to the test meals using a computer-generated random allocation sequence. Participants were asked to consume meals within 20 min. No other food or drink was allowed during the 3 h period on the test days. Following the screening, eligible subjects were advised to refrain from any vigorous physical activity and alcohol intake 24 h before the test day and asked not to consume a high calorie (rich in carbohydrates and fat) meal the night before the test day. On the test morning, subjects reported to the laboratory after an overnight fast of at latest 12 h. They were also asked to collect a 24-h record of their previous day's food intake. This dietary information was processed through FoodWorks Version: 8.0.3551 (Xyris Software (Australia) Pty Ltd.,) to check whether their dietary energy and macronutrients intakes remained unchanged during the study period. The participants' physical activity was assessed on the first test day using the International Physical Activity Questionnaire (long form) to capture the frequency, duration, and intensity of physical activity undertaken during the previous 7 days (17).

### Outcome Measures

Medical history and demographics were recorded and BMI and body composition (was determined using bioelectrical impedance, InBody 230, Biospace Co., Ltd., Seoul, Korea) were determined on the first visit day. Venous blood samples were collected by an in house certified phlebotomist at 0 and 180 min post ingestion of the meal. Hunter Area Pathology Service (Local area pathology service provider, Newcastle, Australia) analyzed the blood samples for fasting and postprandial serum insulin, triglycerides and glucose concentrations. The GlucoTRIG value (16) for each meal was calculated using the formula: (plasma triglycerides_180min_ × plasma insulin_180min_) – (plasma triglycerides_0min_ × plasma insulin_0min_).

### Statistical Analysis

Data are presented as mean ± SEM or median (inter-quartile range, IQR) for continuous variables and, number (*n*) and percentage (%) for categorical variables. The normality of the data was assessed after plotting histograms and by applying the Shapiro-Wilk test. The differences between the groups for continuous variables was tested using Student's *t*-test, chi-square for categorical variables and the Wilcoxon signed-rank test for variables without a normal distribution. Between-group comparisons were analyzed using repeated-measures analysis of variance (ANOVA), and the Bonferroni *post-hoc* test for multiple comparisons or the Kruskal-Wallis test. For all of the statistical analyses, *p*-value < 0.05 was considered to be significant. Statistical analysis was performed using the STATA version 14.1 (StataCorp, Texas, USA).

## Results

### Characteristics, Physical Activity and Dietary Intakes of Subjects

**Figure 1:**
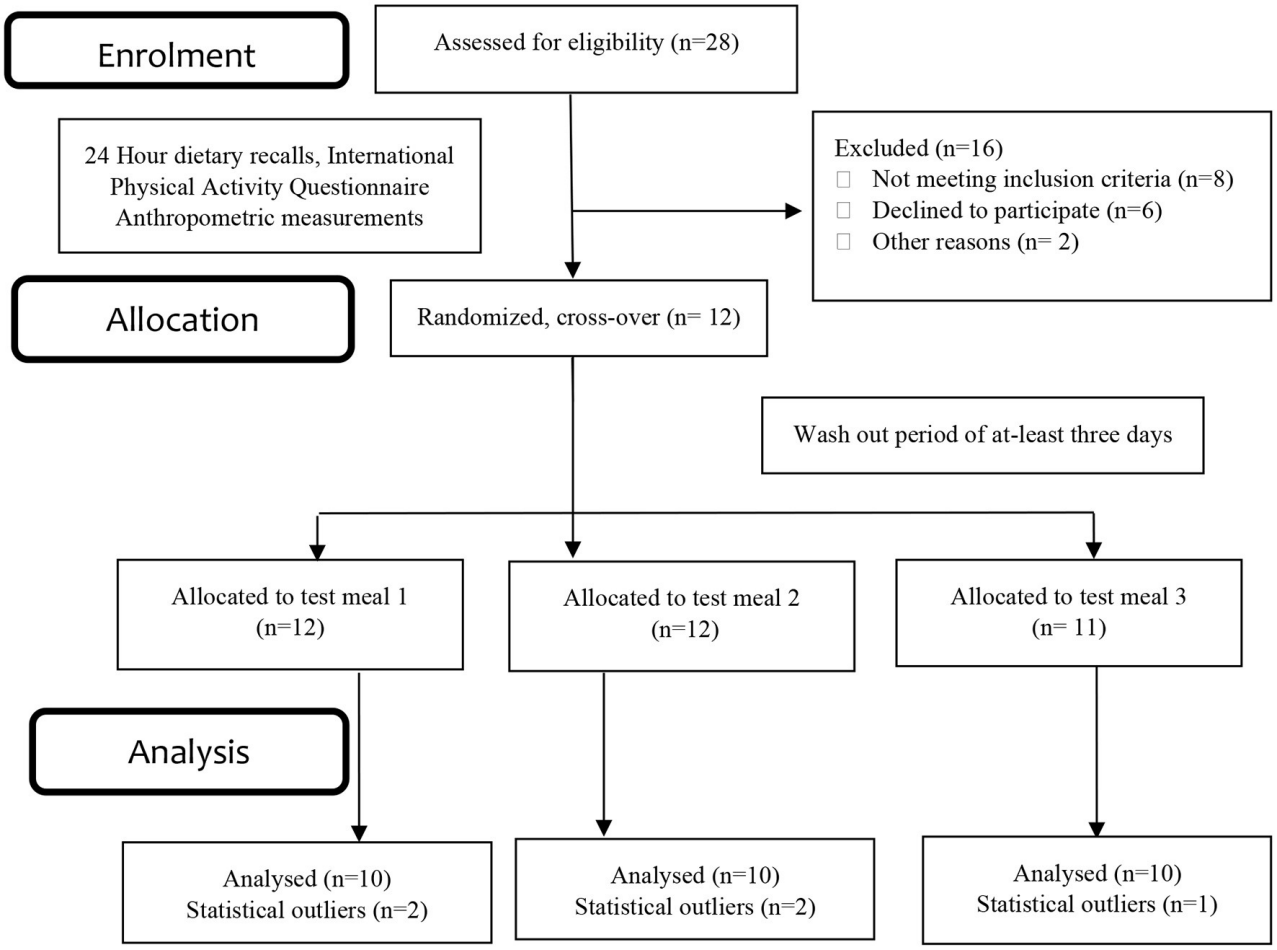
Consolidated standards of reporting trials (CONSORT) flow chart– trial protocol.

The baseline characteristics of the participants have been published previously (16). The results presented in this trial included ten healthy subjects (both females and males) aged 26.7 ± 1.2 years with a BMI of 23.4 ± 0.4 kg/m^2^ (Figure 1). Physical activity analysis indicated seven participants with low physical activity and three with moderate physical activity levels at the baseline visit. There were no significant changes observed in the macronutrient (carbohydrate, protein, total fat) and fiber intakes for the 24 h period before each test day (Table 2). Fasting glucose (4.5 ± 0.1 mmol/L), insulin (41.9 ± 2.9 pmol/L) and triglyceride (0.94 ± 0.1 mmol/L) concentrations were also in a healthy range.

**Table 2:** Predicted energy and macronutrient intakes of the participants 24 h before the test days.

	**Test meals**		
	**Visit 1**	**Visit 2**	**Visit 3**
	***n*** **=** **10**	***n*** **=** **10**	***n*** **=** **10**
**Energy (Kj)**	7656 ± 383.5	6757 ± 661.7	6442.4 ± 375.2
**Carbohydrate (g)**	165 ± 19.0	140 ± 11.5	132 ± 12.1
**Total sugars (g)**	50 ± 10.7	48 ± 4.4	57 ±5.6
**Starch (g)**	115 ± 11.6	93 ± 10.1	77 ± 13.2
**Total fat (g)**	87 ± 8.5	78 ± 12.4	69 ± 7.3
**Saturated fat (g)**	27 ±2.5	26 ±4.1	28± 3.8
**Polyunsaturated fat (g)**	17 ± 3.4	11 ± 2	9 ± 1.3
**Monounsaturated fat (g)**	37 ± 4.3	35 ± 6.7	26 ± 3.0
**Cholesterol (g)**	268 ± 62.5	240 ± 56.8	290 ± 30.6
**Dietary fiber (g)**	22 ± 2.4	21 ± 2.2	19 ± 1.6
**Protein (g)**	85 ± 9.2	81 ± 9.0	88 ± 10.2

*Values are reported as means ± SEM. n represents the number of subjects*.

### Postprandial Insulin, Glucose and Triglyceride Concentrations

Insulin responses to the meals were significantly different (*p* = 0.0129). The mean change in the insulin levels from baseline to 180 min was significantly (*p* = 0.001) lower for M3 [39.6 (27.0)], compared to that for M1, [3.1(17.4)] (Table 3). Although the mean change in the insulin levels for M2 (4.3 ± 1.75) was notably different from the other two meals, it was not statistically significant from that for the other two meals. There were no significant differences (*p* = 0.3673) in postprandial plasma glucose levels at 180 min between the three meals. Mean changes in the triglyceride responses to the meals were not significantly different (*p* = 0.3699) (Table 3).

**Table 3:** Differences in plasma parameters between the meal groups.

**Parameters**	**Meal 1**	**Meal 2**	**Meal 3**	***P-value***
	**(*n* = 10)**	**(*n* = 10)**	**(*n* = 10)**	
Fasting glucose (mmol/L)	4.4 ± 0.1	4.6 ± 0.1	4.6 ± 0.1	0.3206
Glucose (mmol/L) (180 min)	4.0 ± 0.2	4.0 ± 0.2	3.8 ± 0.1	0.7492
Δ Glucose (180–0 min)	−0.4 ± 0.2	−0.5 ± 0.1	−0.7 ± 0.1	0.3673
Fasting insulin (pmol/L)	37 ± 4.9	44 ± 4.8	45 ± 5.1	0.4723
Insulin (pmol/L) (180 min)	90 ± 16.7	73 ± 14.3	46 ± 4.9	0.0888
Δ Insulin (180–0 min)	39.6 (27.0)^a^	20.1 (63.8)	3.1 (17.4)^a^	0.0129
Fasting triglycerides (mmol/L)	0.7 (0.3)	0.67 (0.4)	0.77 (0.6)	0.2827
Triglycerides (180 min) (mmol/L)	1.1(0.8)	0.9(0.6)	0.86 (0.7)	0.6165
Δ Triglycerides (180–0 min)	0.4 (0.8)	0.2 (0.3)	0.1 (0.2)	0.2828

*Data are presented as mean ± SEM or median (IQR) as appropriate. The superscripts represent a significant difference between the medians of two groups*.

a *Δ Insulin is significantly different between meal 1 and meal 3 (P-value - 0.001)*.

The GlucoTRIG value for the reference meal was measured over three different visits in our previous study (16). There were no statistically significant (*P* = 0.2303) differences observed between the GlucoTRIG values for the three visits (16). The GlucoTRIG value for the reference meal obtained from the three mean values was found to be 19 ± 3.5 (16).

### GlucoTRIG Values of the Test Meals

**Figure 2:**
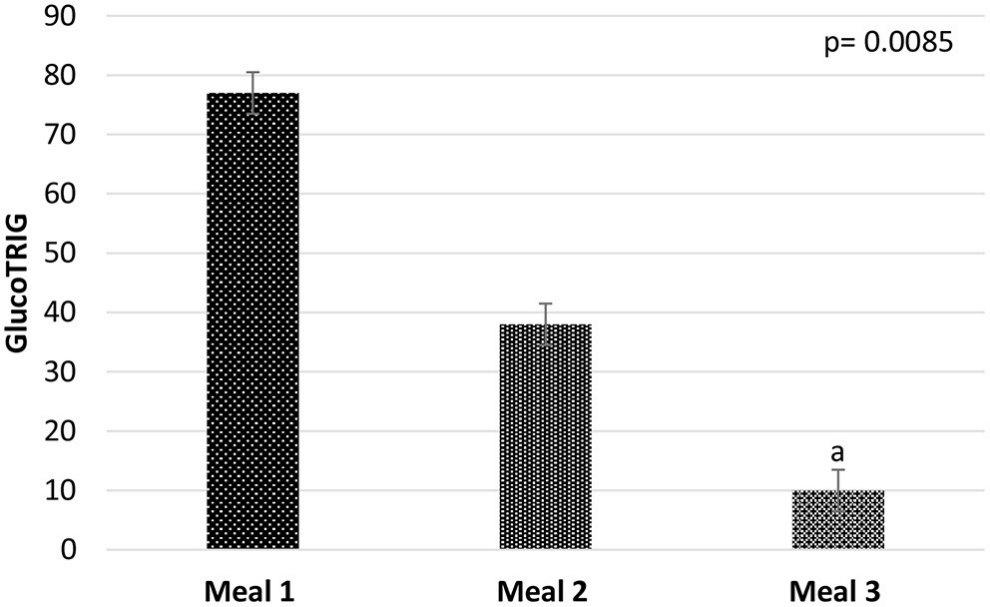
Mean GlucoTRIG values of the meals M1 (*n* = 10), M2 (*n* = 10), and M3 (*n* = 10). Data presented as mean ± SEM. *P*-value represents the repeated measures of ANOVA between the meal groups in a model. ^a^GlucoTRIG value of Meal 3 is significantly differnet from meal 1 (*p* = 0.012).

There were significant (*P* = 0.0085) differences among the test meal groups for the mean GlucoTRIG (*n* = 10) response (Figure 2). Subjects in the M3 group (watermelon, carrot, beans and yogurt) gave the lowest GlucoTRIG response (10 ± 7.7) compared to the other two meals M1 (77 ± 19.8) and M2 (38 ± 12.1). However, the difference in the GlucoTRIG value was significant (*p* = 0.012) only between the M1 and M3 groups. No significant differences were observed between M2 and the other two meal groups.

### Predictors of GlucoTRIG

We assessed the overall extent to which input variables predict GlucoTRIG responses using linear regression. Unadjusted regression analysis indicated the test meals as a significant predictor of GlucoTRIG Values. The significant effect of the test meals (Unadjusted *r*^2^-0.6545; adjusted *r*^2^-0.4433; *p* = 0.0162) remained even after adjusting for input variables: age, sex, fasting triglycerides, fasting glucose, fasting insulin, habitual diet factors (from 24 h food recall), and physical activity levels.

## Discussion

In this study, we report that M1 comprising low glycemic foods exhibited a high GlucoTRIG value. Similarly, M3 with relatively higher glycemic index foods induced a substantially lower postprandial insulin and triglyceride response and thus GlucoTRIG value despite being high in carbohydrate, fiber and protein content as well as containing less fat and minimally processed foods than the other two meals. Both M1 (GlucoTRIG value−77) and M2 (GlucoTRIG value−38) also exhibited a reasonably higher GlucoTRIG response in comparison to the reference meal (GlucoTRIG value−19). Regression analysis indicates that the test meal effect was the sole predictor of GlucoTRIG response out of the several examined variables. The meal effect remained significant even after adjusting for confounding variables such as background diet, age, physical activity levels, and sex. Collectively, the findings indicate that GlucoTRIG (by incorporating both the carbohydrate and lipid responses) may be an improved dietary index for determining a meals' healthiness.

Recent evidence from the Food4Me European randomized controlled trial indicates that the dip in blood glucose levels between 2 and 3 h post meal consumption, but not the glucose rise (0–2 h), or total glucose (iAUC_0−2hr_) is linked to hunger and subsequent food intake (18). The Glucose dip at 2–3 h is also inversely correlated with fasting insulin levels (18). Conventionally, the glycaemic response is measured at 2 h, but it is believed to be insufficient to study postprandial glucose responses (18, 19). Nuclear magnetic resonance spectroscopy analysis (19) recommends three to 4 h as the optimal duration to study post-meal plasma glucose and insulin changes required to maintain glucose homeostasis. GlucoTRIG utilizes a 3-h time point to capture the postprandial insulin response of the meal, which exists around the same time as the dip in the glucose levels and changes in the meal induced metabolic responses. Hyperinsulinemia has been shown to be a significant a etiological factor in obesity, T2DM, and CVD (20). Different insulin patterns were previously evaluated in response to a glucose load (100 g) administered over 3–5 h, with plasma insulin levels assessed from baseline to 180 min (21). With normal insulin tolerance, the peak insulin production occurs between 30 and 60 min, followed by a return to the fasting range at 120 or 180 min (21). Patterns involving prolonged exposure of plasma insulin and a failure to return to basal level have been implicated in the development of insulin resistance and type 2 diabetes (22). Post-prandial analysis from 1,002 healthy adults indicated that a rise in C-peptide (a surrogate marker for insulin) is predictive of a 10-year atherosclerotic CVD risk score (2). These observations strengthen the argument for the necessity to study the insulin responses of the foods and meals, in addition to the postprandial glucose and triglyceride responses.

A series of systematic reviews and meta-analyses of prospective studies reporting on indicators of carbohydrate quality and non-communicable disease showed that higher intake of dietary fiber is associated with a reduction in mortality and the incidence of a wide range of non-communicable diseases and their risk factors (23). Based on this evidence it was recommended to increase dietary fiber to at least 25–29 g per day, with additional benefits likely to accrue with greater intakes (23). This study suggested that dietary GI or glycaemic load might be less sound as an overall measure of carbohydrate quality than dietary fiber and whole grains (23). Results from the current study shows that the meal with highest fiber content (20 g) elicited the lowest GlucoTRIG response compared to meals with relatively lower amounts of fiber. Thus, applying GlucoTRIG may provide additional information over the existing indices in terms of overall meal quality and health outcomes.

There is an increasing health-related concern over the addition of fat with the aim of lowering the glycaemic response of high carbohydrate diets. Fat enrichment to overcome the undesirable effects of some high carbohydrate diets may have undesirable effects on plasma lipids with unfavorable outcomes for weight management. Contrastingly a meta-analysis has reported that reducing total fat intake led to a small but statistically significant and clinically meaningful reduction in body weight (24). The DIETFITS randomized controlled trial indicated similar average weight loss between a healthy low-fat or a healthy low-carbohydrate diet for 12 months, with favorable changes in the lipid parameters in both groups (25). Besides fasting triglycerides, a postprandial rise in circulating triglycerides has been recognized as an independent risk factor for developing CVD and other chronic diseases (23). Studies have also shown that triglycerides peak at 3–4 h post-meal consumption (24). Prolonged postprandial lipemia is implicated in resulting in the formation of highly atherogenic small and dense low density lipoprotein particles and reduced high-density lipoprotein levels, thus increasing disease risk (5). Excess fat intake can induce a lipotoxic state, involving activation of various inflammatory pathways and accumulation of fatty acid metabolites in the insulin-sensitive tissues such as skeletal muscle (26–28). In the current study we observed higher GlucoTRIG responses to meals with higher fat content. Thus, GlucoTRIG overcomes the current limitation of the nutrition grading models by considering the 3-h postprandial lipid response in addition to the glycaemic response.

Studies to date have primarily focused on postprandial 2 h glucose levels for assessing the healthiness of most food groups/meals. Although methodologically superior, post-prandial glucose responses are not reflective of the changes in the postprandial triglyceride response. Relying on glucose alone ignores the effects of protein and fat. Repeated testing of the same meal in different study visits could potentially minimize this inter-individual variation to some extent. Categorizing foods and meals based on a physiological response to both glucose and fat could be a practical approach to promoting health, improving consumers' understanding of healthier foods, and preventing diet-related diseases [10]. Foods with low postprandial glycaemic and lipaemic responses may potentially assist in the prevention of weight gain and the further development of chronic metabolic disorders. GlucoTRIG, capturing postprandial glycaemic and lipaemic responses, could be of both practical and clinical significance in preventing chronic diet-related disease conditions. Moreover, a mixed meal containing a combination of macro-nutrients as a comparator, instead of a single nutrient challenge, would provide a real-life setting for testing meals for health. GlucoTRIG utilizing a 3-h time point instead of a 2-h response, may provide more physiological insight by capturing both the peak triglycerides and residual insulin levels in response to meals.

Including triglycerides and insulin might provide valuable information in regards to the health benefits of the meals. However, at this stage, the measurement of postprandial glucose is methodologically superior to insulin and triglycerides. The assessment of insulin is more costly compared to glucose and methodological discrepancies still exist in relation to measurement of plasma insulin. A recent study also indicates post-meal insulin (c-peptide) secretion can be influenced by the gut microbiome, background serum glycaemic markers and other serum markers (2). In addition, fasting serum lipid markers, glycaemic markers, anthropometric measurements and meal composition were key determinants of the 6-h post meal triglyceride response (2). Although our study indicates that the meal itself is the key determinant of the GlucoTRIG response, factors such as fasting lipid levels, gut microbiome, age, body composition, and sex could potentially be confounding variables.

In the current study we have accounted for only vigorous activity 24 h before the test. Studies in adults have shown that even acute moderate-intensity exercise performed (~24 h) prior to a high-fat meal reduce postprandial TG responses (29–31). Further studies assessing GlucoTRIG for foods and meals, should consider both acute and chronic physical activity levels (both moderate and vigorous) to reduce confounding effects of physical activity on postprandial lipemia. Further research, with the help of technological advances, such as genomic analysis, is required to improve the GlucoTRIG model by accounting for other factors. The clinical benefits of GlucoTRIG also need to be established and this will require adequately powered longitudinal studies. The results of this study are novel but preliminary, and we hope they will stimulate discussion and further research in the area. Further evaluation of GlucoTRIG under different physiological conditions and assessment for test meals is required to establish GlucoTRIG as a food/meal ranking tool. GlucoTRIG has already been shown to be discriminatory in a mixed meal context, but additional studies are needed to determine whether the GlucoTRIG index is useful, and reproducible for single foods. Studies determining the relationship between the fate of absorbed macronutrients and GlucoTRIG values may provide further insights.

## Data Availability Statement

The raw data supporting the conclusions of this article will be made available by the authors, without undue reservation.

## Ethics Statement

The studies involving human participants were reviewed and approved by The University of Newcastle Human Research Ethics Committee (H-2016-0315). The patients/participants provided their written informed consent to participate in this study.

## Author Contributions

RT, PM, HS, and MG designed the study. RT conducted the study, performed data analysis, and drafted the manuscript, which was later revised by PM, HS, and MG. MG had the primary responsibility for final content. All authors read and approved the final content.

## Funding

This project was supported by the Centers of Research Excellence (CoRE) Fund from the New Zealand Tertiary Education Commission.

## Conflict of Interest

The authors declare that the research was conducted in the absence of any commercial or financial relationships that could be construed as a potential conflict of interest.

## Publisher's Note

All claims expressed in this article are solely those of the authors and do not necessarily represent those of their affiliated organizations, or those of the publisher, the editors and the reviewers. Any product that may be evaluated in this article, or claim that may be made by its manufacturer, is not guaranteed or endorsed by the publisher.

## References

[1] Franco-Arellano B Gladanac B Labonté M-È Ahmed M Poon T L'Abbé MR. Nutrient profile models with applications in government-led nutrition policies aimed at health promotion and non-communicable disease prevention: a systematic review Adv Nutr. (2018) 9:741–88. 10.1093/advances/nmy045 30462178 PMC6247226

[2] Berry SE Valdes AM Drew DA Asnicar F Mazidi M Wolf J. Human postprandial responses to food and potential for precision nutrition. Nat Med. (2020) 26:964–73. 10.1038/s41591-020-0934-0 32528151 PMC8265154

[3] Blaak EE Antoine J-M Benton D Björck I Bozzetto L Brouns F. Impact of postprandial glycaemia on health and prevention of disease. Obes Rev. (2012) 13:923–84. 10.1111/j.1467-789X.2012.01011.x 22780564 PMC3494382

[4] Ning F Tuomilehto J Pyörälä K Onat A Söderberg S Qiao Q. Cardiovascular disease mortality in Europeans in relation to fasting and 2-h plasma glucose levels within a normoglycemic range. Diabetes Care. (2010) 33:2211–6. 10.2337/dc09-2328 20424221 PMC2945162

[5] Miller M Stone NJ Ballantyne C Bittner V Criqui MH Ginsberg HN. Triglycerides and cardiovascular disease. Circulation. (2011) 123:2292–333. 10.1161/CIR.0b013e3182160726 21502576

[6] Greenwood DC Threapleton DE Evans CEL Cleghorn CL Nykjaer C Woodhead C. Glycemic index, glycemic load, carbohydrates, and type 2 diabetes. Systematic review and dose–response meta-analysis of prospective studies. Diabetes Care. (2013) 36:4166–71. 10.2337/dc13-0325 24265366 PMC3836142

[7] Shafaeizadeh S Muhardi L Henry CJ van de Heijning BJM van der Beek EM. Macronutrient composition and food form affect glucose and insulin responses in humans. Nutrients. (2018) 10:188. 10.3390/nu10020188 29419785 PMC5852764

[8] Wuesten O Balz CH Bretzel RG Kloer HU Hardt PD. Effects of oral fat load on insulin output and glucose tolerance in healthy control subjects and obese patients without diabetes. Diabetes Care. (2005) 28:360–5. 10.2337/diacare.28.2.360 15677793

[9] Wolpert HA Atakov-Castillo A Smith SA Steil GM. Dietary fat acutely increases glucose concentrations and insulin requirements in patients with type 1 diabetes: implications for carbohydrate-based bolus dose calculation and intensive diabetes management. Diabetes Care. (2013) 36:810–6. 10.2337/dc12-0092 23193216 PMC3609492

[10] Rietman A Schwarz J Tomé D Kok FJ Mensink M. High dietary protein intake, reducing or eliciting insulin resistance? Eur J Clin Nutr. (2014) 68:973. 10.1038/ejcn.2014.123 24986822

[11] Ercan N Gannon MC Nuttall FQ. Effect of added fat on the plasma glucose and insulin response to ingested potato given in various combinations as two meals in normal individuals. Diabetes Care. (1994) 17:1453–9. 10.2337/diacare.17.12.1453 7882816

[12] Fuehrlein BS Rutenberg MS Silver JN Warren MW Theriaque DW Duncan GE. Differential metabolic effects of saturated vs. polyunsaturated fats in ketogenic diets. J Clin Endocrinol Metab. (2004) 89:1641–5. 10.1210/jc.2003-031796 15070924

[13] Vessby B Uusitupa M Hermansen K Riccardi G Rivellese AA Tapsell LC. Substituting dietary saturated for mono-unsaturated fat impairs insulin sensitivity in healthy men and women: the KANWU Study. Diabetologia. (2001) 44:312–9. 10.1007/s001250051620 11317662

[14] Fryk E Olausson J Mossberg K Strindberg L Schmelz M Brogren H. Hyperinsulinemia and insulin resistance in the obese may develop as part of a homeostatic response to elevated free fatty acids: a mechanistic case-control and a population-based cohort study. EBioMedicine. (2021) 65:103264. 10.1016/j.ebiom.2021.103264 33712379 PMC7992078

[15] Dias CB Moughan PJ Wood LG Singh H Garg ML. Postprandial lipemia: factoring in lipemic response for ranking foods for their healthiness. Lipids Health Dis. (2017) 16:178. 10.1186/s12944-017-0568-5 28923057 PMC5604516

[16] Thota RN Moughan PJ Singh H Garg ML. GlucoTRIG: a novel tool to determine the nutritional quality of foods and meals in general population. Lipids Health Dis. (2020) 19:83. 10.1186/s12944-020-01268-w 32366255 PMC7199359

[17] Craig CL Marshall AL Sjöström M Bauman AE Booth ML Ainsworth BE. International physical activity questionnaire: 12-country reliability and validity. Med Sci Sports Exerc. (2003) 35:1381–95. 10.1249/01.MSS.0000078924.61453.FB 12900694

[18] Celis-Morales C Livingstone KM Marsaux CF Macready AL Fallaize R O'Donovan CB. Effect of personalized nutrition on health-related behaviour change: evidence from the Food4Me European randomized controlled trial. Int J Epidemiol. (2017) 46:578–88. 27524815 10.1093/ije/dyw186

[19] Taylor R Magnusson I Rothman DL Cline GW Caumo A Cobelli C. Direct assessment of liver glycogen storage by 13C nuclear magnetic resonance spectroscopy and regulation of glucose homeostasis after a mixed meal in normal subjects. J Clin Invest. (1996) 97:126–32. 10.1172/JCI118379 8550823 PMC507070

[20] Shanik MH Xu Y Škrha J Dankner R Zick Y Roth J. Insulin Resistance and Hyperinsulinemia. Is hyperinsulinemia the cart or the horse? Diabetes Care. (2008) 31(Suppl. 2):S262–8. 10.2337/dc08-s264 18227495

[21] Kraft JJL. Detection of diabetes mellitus *in situ*. Occult Diabetes. (1975) 6:10–22. 10.1093/labmed/6.2.10

[22] DiNicolantonio JJ Bhutani J Okeefe JH Crofts C. Postprandial insulin assay as the earliest biomarker for diagnosing pre-diabetes, type 2 diabetes and increased cardiovascular risk. Open Heart. (2017) 4:e000656-e. 10.1136/openhrt-2017-000656 29225902 PMC5708305

[23] Reynolds A Mann J Cummings J Winter N Mete E Te Morenga L. Carbohydrate quality and human health: a series of systematic reviews and meta-analyses. Lancet. (2019) 393:434–45. 10.1016/S0140-6736(18)31809-9 30638909

[24] Hooper L Abdelhamid A Moore HJ Douthwaite W Skeaff CM. Summerbell CD. Effect of reducing total fat intake on body weight: systematic review and meta-analysis of randomised controlled trials and cohort studies. BMJ. (2012) 345:e7666. 10.1136/bmj.e7666 23220130 PMC3516671

[25] Gardner CD Trepanowski JF Del Gobbo LC Hauser ME Rigdon J Ioannidis JPA. Effect of low-fat vs low-carbohydrate diet on 12-month weight loss in overweight adults and the association with genotype pattern or insulin secretion: the DIETFITS randomized clinical trial. JAMA. (2018) 319:667–79. 10.1001/jama.2018.0245 29466592 PMC5839290

[26] Norata GD Grigore L Raselli S Redaelli L Hamsten A Maggi F. Post-prandial endothelial dysfunction in hypertriglyceridemic subjects: molecular mechanisms and gene expression studies. Atherosclerosis. (2007) 193:321–7. 10.1016/j.atherosclerosis.2006.09.015 17055512

[27] Shin HK Kim YK Kim KY Lee JH Hong KW. Remnant lipoprotein particles induce apoptosis in endothelial cells by NAD(P)H Oxidase mediated production of superoxide and cytokines via lectin-like oxidized low-density lipoprotein receptor-1 activation. Circulation. (2004)109:1022–8. 10.1161/01.CIR.0000117403.64398.53 14967724

[28] Ting HJ Stice JP Schaff UY Hui DY Rutledge JC Knowlton AA. Triglyceride-rich lipoproteins prime aortic endothelium for an enhanced inflammatory response to tumor necrosis factor. Circ Res. (2007) 100:381–90. 10.1161/01.RES.0000258023.76515.a3 17234968

[29] Lee S Burns SF White D Kuk JL Arslanian S. Effects of acute exercise on postprandial triglyceride response after a high-fat meal in overweight black and white adolescents. Int J Obes. (2013) 37:966–71. 10.1038/ijo.2013.29 23507997 PMC4035105

[30] Petitt DS Cureton KJ. Effects of prior exercise on postprandial lipemia: a quantitative review. Metabolism. (2003) 52:418–24. 10.1053/meta.2003.50071 12701052

[31] Plaisance EP Mestek ML Mahurin AJ Taylor JK Moncada-Jimenez J Grandjean PW. Postprandial triglyceride responses to aerobic exercise and extended-release niacin. Am J Clin Nutr. (2008) 88:30–7. 10.1093/ajcn/88.1.30 18614721

